# Understanding Levels and Motivation of Physical Activity for Health Promotion among Chinese Middle-Aged and Older Adults: A Cross-Sectional Investigation

**DOI:** 10.1155/2019/9828241

**Published:** 2019-08-25

**Authors:** Md Mizanur Rahman, Chang Yong Liang, Dongxiao Gu, Yong Ding, Monira Akter

**Affiliations:** ^1^School of Management, Hefei University of Technology, Hefei 230009, China; ^2^Key Laboratory of Process Optimization and Intelligent Decision-Making, Ministry of Education, Hefei 230009, China; ^3^The School of Informatics, Computing and Engineering, Indiana University Bloomington, Bloomington, IN 47405-3907, USA

## Abstract

**Objective:**

Middle-aged and older Chinese adults (35 to 75) failed to meet the recommendations of physical activity guidelines for health promotion, because of a lack of understanding of their perspective on physical activity. This study considers the physical activity levels and motivation among middle-aged and older Chinese adults partitioned into three different participation groups (sports, exercise, and recreational and cultural activities).

**Methods:**

A cross-sectional analysis was performed on 633 participants, based on two different levels of physical activity levels. The International Physical Activity Questionnaire (IPAQ) and the Exercise Motivation Inventory (EMI-2) with 14 subscales were used to measure the participant's physical activity levels and physical activity motivation for the three different group activities.

**Results:**

Results indicate those participants' physical activity levels and motivation varied significantly among the different groups. The participants who engage in recreational and cultural activities have a higher motivation for physical activity, as compared with those whose primary form of physical activity is sports and exercise, and a higher probability to fulfill the global recommendations concerning physical activity. Participants who used recreational and cultural activities as their major type of physical activities were more motivated by “intrinsic” aspects. In contrast, those who participate in sports and exercise as their main type of physical activity are more motivated by “extrinsic” aspects.

**Conclusion:**

Close friends and family members of one's home and community have potential influence in physical activity and recreational and cultural activity participants are highly motivated and luckier than others to fulfill the goal of physical activity levels.

## 1. Introduction

Encouraging ordinary participation in physical activity (PA) is a global public health priority [1, 2] of the World Health Organization (WHO). According to WHO [3], 26% male and 35% female were doing less physical activities in high-income countries than 12% male and 24% female in low-income countries. WHO has provoked the member nations to develop policies and programs which promote physical fitness through diet and PA in peoples' daily life. WHO [1] defined physical activity as “as any bodily movement produced by skeletal muscles that requires energy expenditure.” In this study, we proposed physical activity as “an activity to keep a person body fitness, making strong muscles, as well as psychologically strong.” To participate in structured physical exercise or sports means to be active throughout the day and avoiding long periods of inactivity. WHO highlights the worth of daily activities, such as playing games, sports, exercise, dance, gardening, and walking or cycling; see “Global Recommendations on Physical Activity for Health” [3].

Previous studies [4–7] investigated the motivations for sports or exercise involvement [8]. Kilpatrick et al. [6] conducted a study among college students in the USA to examine their participation motive towards sports vs. exercise. Their findings backed that intrinsic motivations like affiliation, competition, challenge, and enjoyment were ranked higher in sports than for exercise. Their study also supported that external motivations such as health and appearance were the prime motivations to participate in exercise.

Laziness and inactivity have been acknowledged and seen as a high-risk aspect for illness. 12%–19% of the risk linked with major NCDs (noncommunicable chronic diseases) [9, 10] has been raised in China [3, 11]. According to studies from the China Health and Nutrition Survey, the overall level of PA among Chinese people gradually declined over the last two decades; for example, the metabolic equivalent hours per week (MET-h/week) among males decreased from 382 to 264, while decreasing from 420 to 243 for females [12].

Existing studies about physical activities among middle-aged and older Chinese people are scarce and also limited in quality and scope. In particular, there is a lack of knowledge about the patterns and prevalence of PA (exercise, sports, and participation into recreational and cultural activities (RCAs)) among middle-aged and older peoples in urban areas. The lack of advanced studies in this area is one of the most important hindrances towards the development of strategies for addressing the significance of physical activities to increase PA rate in China. Our study helps to bridge this gap; it presents how physical activities significantly influence and motivate the life of middle-aged and older Chinese people, with a key focus on people living in urban areas of China. The study has significant public health implications for scholars, physician, and policymakers for future studies.

### 1.1. Conceptualization of Physical Activity (Exercise vs. Sports vs. RCAs)

“Physical activity” is a collection of complex activities which include bodily functions or events involving skeletal motions, with the aim of spending the energy accumulated throughout the prior activities of the day [13]. In addition, it also covers daily family activities like grocery shopping, household chores, or taking the trash out.

“Exercise” is a part of PA [14], which is defined as a subsection of spare time related to PA and is structured, designed, and recurring on a daily basis or otherwise to enhance physical condition and body fitness [15]. In particular, this includes flexibility and body harmony, as well as balance, cardiovascular fitness, brawny fitness, and skill-related components of fitness associated with enhanced performance between middle-aged and older adults [1, 16]. The key purpose of doing exercises such as jogging, running, cycling, calisthenics, water aerobics, and weight-lifting training is to maintain or improve one's physical condition.

The concept of “sports” is not succinct [17]. Therefore, sports include entire forms of spirited PA or events which take place in an organized or casual manner. The key motive is to utilize them in order to maintain and develop further physical capabilities and skills. In addition to providing satisfaction to the participants, it also procures amusement for the audience. Owing to the motive of this inquiry, sports are prearranged events which individuals, as well as groups of individuals, engage in for their benefit and improve their skills at these sports [17]. For instance, our study considers basketball, badminton, tennis, table tennis, golf, racquetball, and soccer.

*The Park and Recreation Professional's Handbook* defines recreation as “an activity that people engage in during their free time, which people do enjoy and recognize as having socially compensative values” [18]. Recreation actions consist of PA for individuals, or groups of individuals, done during their spare time and procuring satisfaction. Recreation participants take on special activities for pleasure according to their preferences and circumstances. Some examples of recreational activities are mountaineering, dancing, fishing, farming, and hiking, to name a few.

Also, “culture” comprises social behaviors and norms observed in societies or human communities [19]. Several aspects of human activities and community practices, e.g., music, dance [20], painting, cultural celebration, religion and tool usage, food preparation, livelihood, and fashion, are universal as they are found in every human community. Chinese culture (中华文化; pinyin: Zhōnghuá wénhuà) [21] is one of the world's most ancient cultures where verbal communication, ceramic objects, structural design, dance, music, literature, martial arts, cuisine, visual arts, ways of life, political principles, and history have a profound impression on the world. In this study, we consider dance, dance exercise, thaichi (太极), wushu (武术), and martial arts as recreational and cultural activities (RCAs).

### 1.2. Self-Determination Theory

According to Deci and Ryan [22] and Ryan et al. [23], self-determination theory (SDT) is “a macro theory of human motivation (intrinsic and extrinsic) and personality that concerns people's inherent growth tendencies and innate psychological needs.” The authors propose that the most favorable purpose is to enjoy through psychosomatic desires [24] of connectedness, ability, and independence. Previous studies show that there is a relationship among sports activities with intrinsic motivations and exercise with extrinsic motivations. We have selected SDT for this study, as it relates to motivation and, through SDT, we can achieve our objectives. In this study, we considered sports and RCAs as the part of intrinsic motivation, while exercise is viewed as the part of extrinsic motivation. We defined intrinsic motivation as the inner willingness to do physical activities and extrinsic motivation as the willingness to perform physical activities driven by others.

### 1.3. Physical Activity Motivation

To promote PA, we first consider the motives behind local people's involvement in physical activities like exercise or sports or RCAs. Also, the kinds of sports, exercise, and recreational activities modify participation motives [25]. Research studies have perused exercise motivation along with a multiplicity of populations, inclusion primary school [26] adolescents [27], college-age students [6], university students [5], adults [4, 28], middle-aged adults [29], and older adults [30].

In [28], it is found that competence and enjoyment are the major motivations for energetic and active individuals who differentiate their elementary PA as either “individual sports” or “exercise/fitness activities” or “individual cultural activities.” However, Kilpatrick et al. [6] found that exercise is habitual body-related motivation and that participants are encouraged to practice various sports as a way to keep exercising.

Recreational styles of PA, i.e., RCAs, might be more intimately connected to enviable motivational techniques than to individual [24] motivation. This will possibly improve the assessment of rates to energetic free time activities through aerobic or muscle-based involvements [31]. PA could be changing from controlled, acute aerobic exercise to less structured prearranged and extra routine-oriented types of exercise, like dance, dance exercise, thaichi, wushu, and martial arts.

## 2. Methods

### 2.1. Measure

The survey questionnaire employed to gather data had two components: (i) demographic and general characteristics and (ii) constructs based on the adapted edition of “EMI-2” (Exercise Motivation Inventory 2), which is physical activity behavior by using measures from earlier literature and modified according to this study. Each item is measured on a five-point Likert scale anchored by selecting between “strongly disagree” (1) and “strongly agree” (5). The elementary intention of the survey is to evaluate physical activity levels (WHO) and then physical activity motivation to expand the motivational constructs connected through exercise and sports involvement with the additional physical activity type RCAs. Besides, this survey tests the motivational factors (extrinsic or intrinsic) between diverse physical activity benefits (sports, exercise, or RCAs) in middle-aged and older adults (35 to 75+ years old) [28]. The authors are fluent in the local language of the selected country as well as in the English language. They translated the English version of the questionnaire into local languages, and then it was interpreted back into English to check the quality of the translation. This bilingual approach helps ensuring that the translation is accurate and valid. To ensure reliability and internal consistency between the questions associated with each variable, a “Cronbach's alpha (*α*)” test was conducted.

#### 2.1.1. Measurement of Physical Activities

In the survey, participants were provided with clear definitions of exercise, sports, and RCAs. In addition to these definitions, examples were delivered of sports (basketball, badminton, tennis, table tennis, football, golf, racquetball, and soccer), exercise (jogging, running, cycling, hiking, aerobics, water aerobics, and weight-lifting training), and RCAs (dance, dance exercise, thaichi (太极), wushu (武术), and martial arts). Then, they were asked to identify their principal type of PA (exercise, sports, and RCAs) according to the classifications and illustrations supplied. Furthermore, physical activity levels are classified into two categories according to time and activity types (vigorous and moderate). Following clarifications and examples, the participants' responses were checked for reliability to eliminate random answering and item dissimilarities (due to translation) among sports, exercise, and RCAs.

Participants gave information on their weekly PA levels while questioned, “In a typical week, how many hours in total do you spend participating in physical activity?” Reactions are categorized into six different parts (Table 1). Participants are classified into the grouping of having the minimum PA (75 minutes/week), moderate PA (150 minutes/week), and vigorous PA (300 or 300+ minutes/week) requirements.

Participants gave information on their weekly PA levels in response to the question “In a typical week, how many hours/minutes in total do you spend participating in physical activity?” Reactions were categorized into six different segments (Table 1). PA levels were evaluated via the “International Physical Activity Questionnaire (IPAQ)” which still has been accepted to determine the weight of PA and judgmental instrument that has been experienced for reliability and validity [32]. Participants were classified into three activity level requirement groups: minimum PA (75 minutes/week), moderate PA (150 minutes/week), and vigorous PA (300 or 300+ minutes/week).

#### 2.1.2. Measurement of Physical Activity Motivation

For this study, we modified “Exercise Motivation Inventory (EMI-2)” used by Kilpatrick et al. [6] which was initially (EMI) developed by Markland and Ingledew [7]. Though the EMI scale was initially applied to judge motivation to exercise, however, the current research expands the focus to sports, exercise, and RCAs towards PA. EMI-2 is includes 51 questions covering 14 diverse motivational subindexes which consist of Affiliation (AF), Appearance (AP), Challenge (CH), Competition (COM), Enjoyment (EN), Health Pressures (HP), Ill-Health Avoidance (IHA), Nimbleness (NI), Positive Health (PH), Revitalization (RE), Social Recognition (SR), Strength and Endurance (SE), Stress Management (SM), and Weight Management (WM).

### 2.2. Sample Selection and Data Collection

In the questionnaire first part, the participants were asked about their gender, age, marital status, living arrangement, education, self-rated health, social environment motivator, and physical functioning limitations. In the second part, we used that question (“In a typical week, how many hours in total do you spend participating in physical activity?”) and IPAQ to identify participants' PA levels. Thereafter, we used EMI-2 questionnaire to identify participants' PA motivation.

Prior to starting the main survey, first, a study based on a focus group was conducted with few Ph.D. students from the school of management having specialized skills in survey design. Subsequently, minor changes were made regarding the sequence and wording of some questions following recommendations from the focus group. Second, to take the feedback and also to confirm the content's validity with the physical activity system, forty valuable reviews were analyzed after which the questionnaire was considered to be in its final version. After final version, that questionnaire was sent to a different group of people via “web link” and “barcode” in different groups, playground, parks, malls, and recreational centers where middle-aged and old people are engaged in different PA in Hefei City in Anhui Province in China for collecting data. We distributed the questionnaires to middle-aged and older people, who completed it but considered only those filled by participants whose age was above 35 [28].

Most of the respondents were from 55 to 64 years of age, then 65–74 years of age, showing that people from these age groups have more experience of exercise, sports, and RCAs. A total of 786 questionnaires were collected, but 153 of these were rejected because of missing data or were filled by respondents who are not engaged in PA for at least 75 minutes per week. Thus, 633 replies were used for the final study. Participants were informed that involvement was voluntary and they could remove themselves from the survey at any time.

### 2.3. Data Analysis

To check both the measurement of physical activity levels and the motivation, we selected the SPSS 23.0 to analyze the data. Statistical analysis was utilized to find out which particular styles of physical activity are preferred and determine the higher motivations in middle-aged and older people. To compute participants' PA level, we conducted an *F*-test and an “analysis of variance (ANOVA)” for the groups (sports, exercise, and RCAs) assessment. The significance level for alpha was *α* ≤ 0.05. We also evaluated participants' motivation on the basis of the “EMI-2” adapted edition. Applying “multivariate analysis of variance (MANOVA)” with the types of PA (sports, exercise, and RCAs) as the “independent variables” and the 14 subindexes (AF, AP, CH, COM, EN, HP, IHA, NI, PH, RE, SR, SE, SM, and WM) scores as the “dependent variables” for this research proposes [33]. This analysis allowed us to search for the outcome of activity types on the entire motivational scales, where outcomes are considered significant whenever *p* ≤ 0.05.

## 3. Results

The IPAQ short edition, consisting of seven questions, tested the participant's level of physical activity over the previous seven days. Correlation assessments exposed that the “IPAQ” short edition has a Cronbach's alpha (*α*)=0.83. However, our research found alpha (*α*) values for sports (*α*=0.81), exercise (*α*=0.84), and RCAs (*α*=0.87) (Table 1).

The demographics segment contained participants' gender, age, marital status, living arrangement, education, self-rated health, social environment motivator, and physical functioning limitations. Participants were asked about the time spent on physical activity and were categorized into three ranges as minimum PA, moderate PA, or vigorous PA requirements (Table 1). They were also asked whether they are a member or members of any PA club or contributed in any sports or PA competition. Moreover, they were asked to identify their principal type of PA (exercise, sports, and RCAs) according to the classifications and illustrations supplied. Furthermore, physical activity levels are classified into two categories according to time and activity types (vigorous and moderate). Participants specified the number of days they joined for each type of PA and, for each day, how long (time) they participated and then categorized in which PA level it belonged. The number of days multiplied by the quantity of time spent on each PA supplied us with the total quantity of time engaged in PA for all levels. Here, the means of the initial data are used in the statistical assessment of individual PA levels.

From Table 2, information concerning attendees, demographics, and physical condition individuality are dedicated consistent to PA levels. 245 males (38.7%) and 388 females (61.3%) in the bulk part among age range 45–74 (76.6%) years old are mostly married (406; 64.1%), had completed a degree (43.4%), and lived with family members (74.4%).

Respondents who engaged in minimum, moderate, and vigorous PA every week (*n* = 544; 70.1%) normally indicated that they were in “good health” (83.2%, 84.8% vs. 87.6%) and (*n* = 417) have no limitations in physical functioning (27.4%, 54.8% vs. 86.1%) in contrast to individuals who involved in PA less recurrently (*p* < 0.001). Middle-aged and older adults who are engaged in moderate or vigorous PA every week indicated advanced levels of motivations from friends (66 + 97; 86.2%) and family (91 + 178; 89.96%) compared to participants reporting 75 minutes PA per week from friends (13.8%) and family (10.04%).

Contemporary PA levels were observed between contributors who were grouped as being energetic in sports, exercise, and RCAs. Components tested for this decision integrated the participant's vigorous vs. moderate sports, vigorous vs. moderate exercise, and vigorous vs. moderate RCAs time throughout the previous seven days (Table 3). This research reports diversity in PA levels between the groups of sports, exercise, and RCAs. PAs for this study participants included sports (*n* = 117), exercise (*n* = 206), and RCAs (*n* = 310). Descriptive statistics of PA involvement expressed research participants performing vigorous PA 3 days for every week (*M* = 3.58) plus 30–60 minutes per day (*M* = 3.15), and also for participants involved in moderate PA 3 days per week (*M* = 3.83) for 30–60 minutes per day (*M* = 3.51). The group of participants who showed that RCAs are their major form of activity has the highest percentage of participants who engaged in PA in the last 7 days. Moreover, the RCAs involvement group has the highest proportion of individuals who indicated that involvement in at least 6 days of vigorous PA as well as moderate PA aimed for 90+ minutes per day (Table 3). However, the sports participation group reported the lowest participation percentage in PA.

The “ANOVA” test expresses significant PA effects for 9 out of 14 motivation subscales. Research participants indicate superior motivation to participate in PA as “sports” for “Strength and Endurance, Positive Health, and Enjoyment.” Attendees whose major activity is “exercise” point out the better motivation for “Positive Health, Stress Management, Affiliation, and Weight Management.” Among participants whose primary activity is RCAs, the top three motives are “Stress Management, Enjoyment, and Affiliation.” Between PA categories (Table 4), the largest effect size differentiation is for Affiliation. Outcomes of “MANOVA” expressed significant and most important outcomes for PA, where “Wilks's lambda” = 0.77, *F* (14, 633) = 6.629, *p* < 0.001. Outcomes that point out toward motivations vary by PA type (sports, exercise, and RCAs).

Furthermore, we studied statistical segregations between PA styles and we used statistical means into motivational items rankings (Table 4). Motivational item's ranking allows us to assess diverse variations between activity categories. In general, participants were more motivated for engaging in PA for “Stress Management, Enjoyment, Affiliation, and Positive Health.” The “MANOVA” specifies that motivations are different for distinct PA styles. However, few similarities are found among participants motivation to participate in exercise and RCAs according to motivational ranking.

## 4. Discussion and Implications

This study examines the middle-aged and older adults' understanding of PA and social-ecological individuality and their self-reported PA levels. This study is in line with [6, 34] and supports the conception that RCAs styles of PA are significant sources of PA while individuals tend to be better physically energetic than individuals who are far away. Studies on Chinese people's motivation to take part in sports and exercises with additional RCAs are scarce. Results of this study indicate that 48.97% of individuals take part in RCAs (including a majority of middle-aged people) and 32.55% in exercise, while only 18.48% involves themselves in sports activities.

Our primary motive for this study is to apply the “EMI-2” scale to compare the level of motivation involved in exercises, sports, and RCAs. Results of this study confirmed that “Enjoyment” is a usual exposition of “intrinsic motivation,” which makes it more significant in sports and RCAs than in exercise involvement. “Enjoyment” and “Stress Management” are connected with intrinsic motivation, and bodily linked motivations like “Positive Health” and “Weight Management” are more linked to extrinsic motivation.

Results of this study show that the top three types of motivations (out of the fourteen categories of motivations) for exercise participants are “Positive Health, Stress Management, and Affiliation.” The top three motivations towards RCAs are “Stress Management, Enjoyment, and Affiliation.” Finally, the top three motivation types for participants whose primary kind of PA is sports are “Strength and Endurance, Positive Health, and Enjoyment.”

Results of this study are in contradiction with earlier studies [4–7], showing that those who involve themselves in RCAs as their basic types of PA have motivational constructs (Stress Management and Affiliation) which are intrinsic ones. On the other hand, physical condition and fitness purposes are usually extrinsic and have been established to improve exercise activities, in particular when individuals find their relevance in movement and include it as an element of their desire principles and requirements [35]. Similar internalization appears in “exercising” for extrinsic reasons like physical condition and fitness. But physical condition and fitness shift closer to intrinsic motivation and become better liable to be maintained in the long term [36].

An individual whose primary activity is RCAs reports the highest percentage of minutes or days in vigorous PA and moderate PA (Table 3). It emphasizes that there are more participants contributing to a minimum of 6 days of moderate or vigorous PA for ninety-plus minutes every day (Table 3). On the other hand, sports participants report the lowest percentage of participation. This is due to the unavailability of sports or league competition and also to the limitation of playing for amusement for the group of middle-aged and older adults. Nevertheless, they do have the capability to participate on a daily basis to sports activities in their backyard, such as basketball or table tennis.

Engaging in RCAs was more correlated with motives related to Social engagement as well as Enjoyment. Also, Enjoyment and Stress Management are found to be more important in motivating RCAs but to a lesser level in sports and even less so for exercise. While activity is fun and enjoyable with challenges, people willingly engage in activities with playfulness and thereby need no or little extrinsic motivations to do so, in particular, when the activity involves opportunity, enjoyment, a sense of competence, and an opportunity for social relations [23, 27] like in the case of RCAs and sports. Furthermore, we found that participants who are club members of any entertaining or recreational organization or who regularly participate in PA with family members or friends have more chances to achieve high level of physical activity.

### 4.1. Theoretical Implications

This study contributes significantly to the prevailing literature. First, the MANOVA suggested that internal motivational factors (Enjoyment, Ill-Health Avoidance, and Affiliation) exert significant influence on PA intention. Through the results of this study, scholars can perform both intrinsic and extrinsic analyses to accurately evaluate the influence of exogenous variables.

Second, in perspective of the role of PA values, the findings of this study show sports and exercise intention values have a lesser influence on participants PA motivational intentions than RCAs values. However, these outcomes are contrary to those seen in earlier studies [4–7], but this study indicates that RCAs have a direct significant influence on participants Stress Management, Enjoyment, and Affiliation.

Third, contrary to previous studies [4–7] of PA motivations, Affiliation and Enjoyment are found to have a key effect on PA motivational intentions in middle-aged and older Chinese adults. A possible explanation could be that when participants identify RCAs as easy to use, they may develop a positive intention for the effectiveness of the PA. This positive connection could endorse the fact that participants are keen to adopt RCAs on the basis of use. Scholars are recommended to apply these constructs in their research to get more awareness from their target audiences and add new facts to the PA styles.

Finally, RCAs form a new phenomenon in China and tend to be studied along demographic aspects like gender, income, region (e.g., urban or rural), and religion that may affect its adoption. Researchers could develop mobile applications by using artificial intelligence digital tools (like a watch) to guide or monitor how people should do PA in everyday life to maintain better health. With these applications or tools, participants could ask any questions in the national language about a particular method without the difficulty for doing activities.

### 4.2. Managerial Implications

Besides the implications for scholars, this study also suggests several implications for project managers. First, project managers are strongly encouraged to improve their RCAs techniques [37] because this specific aspect has a great significance for the PA of participants who use RCA's platforms. Given that participants are getting used to executing decisions on an “anywhere-anytime” basis, i.e., whenever they get the time, they will do PA. For example, dance exercise has included structural assurances in its PA policy that has made PA successful.

Second, the findings of this study offer handy information for PA decision-makers in China, as the study provides full information on how to use different ways of managing and developing PA strategies. For example, this study confirms that RCAs had a significant influence on middle-aged and older adults PA motivations. Results recommend that participants of RCAs may be more likely to do PA just for fun and enjoyment, whereas effective participants may be motivated to use RCAs to enhance their psychophysical productivity. So, trainer or decision-makers should ensure a good quality of activities and a diversified range of activities and services and other activities related to effective PA values. More precisely, given utilitarian PA values, project managers or decision-makers should offer multipurpose services and free training systems for participants. Moreover, due to cultural beliefs, it becomes easier for Chinese people to go outside and do PA. RCAs (like dance, wushu, and thaichi) provide a platform where the Chinese people can easily do PA in any place where people like with convenience and long-lasting with all norms and cultural values.

Finally, it is vital for PA managers or decision-makers to adopt one of the most attention-grasping trends of PA as RCAs in Chinese people. Chinese people must understand the need to integrate people's PA systems before it is too late. It could assist people to develop optimistic views relating to the PA values. This trend could reshape the whole physical activity sector.

## 5. Limitations and Conclusion

There are some limitations to this research. First, the study framework (single city in single country) may limit the generalization of results from this study. Future studies may focus on different cities or cross-cultural study. Second, gender evaluations were not performed in the sample, but men and women might have diverse motives for sports and exercise involvement [38] and RCAs. Another limitation is that our research is based on cross-sectional motive. Future research should comprise events of actual exercise, sports, and RCAs performance in a time-lagged proposed apropos to establish how participation is influenced and predicted by diverse motives. Finally, information could be confusing if there was lack of understanding definitions of moderate or vigorous activity and any complexity in differentiating types of PA (exercise, sports, and RCAs). Furthermore, upcoming investigations should focus on recreational activities especially dance/dance exercise motivation.

## 6. Conclusion

Attention needs to be paid to PA for middle-aged and older adults. Similar studies and analysis could be extended to a variety of activities with diversity in intensity, formality/informality, and connected altitudes of social communication. Our study discovered that close friends and family members of one's home, and the community, have the potential to influence middle-aged and older adults to participate in PA. The fundamental statement of this research is that RCAs participants might be more personally motivated than those who participate in exercise and sports and they could achieve the goal of PA levels which was proposed by the WHO [1, 2].

## Figures and Tables

**Table 1:** Physical activity per week.

Hours/week	Sports (*n* = 117)	Exercises (*n* = 206)	RCAs (*n* = 310)	Cronbach's alpha (*α*)
1-2	27	38	57	
3-4	54	71	87	
5-6	21	56	96	
7-8	10	31	47	0.83
9-10	3	9	18	
≥11	2	1	5	

*α*	0.81	0.84	0.87	

**Table 2:** Demographics with minimum, moderate, and vigorous levels of physical activities.

Sociodemographic characteristics		Physical activity levels		
		Minimum PA	Moderate PA	Vigorous PA
Total sample 100%	(*N* = 633)	17.9% (113)	31.1% (197)	51.0% (323)

*Gender*				
Male	38.7% (245)	41.6% (47)	33.5% (66)	40.8% (132)
Female	61.3% (388)	58.4% (66)	66.5% (131)	59.2% (191)

*Age*				
35–44	13.1% (83)	13.3% (15)	13.2% (26)	13.0% (42)
45–54	21.8% (138)	21.1% (25)	21.8% (43)	21.7% (70)
55–64	32.2% (204)	31.9% (36)	32.0% (63)	32.5% (105)
65–74	22.6% (143)	23.0% (26)	22.8% (45)	22.3% (72)
75+	10.3% (65)	9.7% (11)	10.2% (20)	10.5% (34)

*Marital status*				
Single/not married	15.5% (98)	20.4% (23)	14.3% (28)	14.5% (47)
Married	64.1% (406)	61.0% (69)	55.8% (110)	70.3% (227)
Widower/widower/divorced	20.4% (129)	18.6% (21)	29.9% (59)	15.2% (49)

*Living arrangement*				
Alone	18.8% (119)	17.7% (20)	19.3% (38)	18.9% (61)
Living with family	74.4% (471)	75.1 (85)	75.1 (148)	73.7% (238)
Living in a community care/nursing home	6.8% (43)	7.1% (8)	5.6% (11)	7.4% (24)

*Education*				
Preliminary education	14.7% (93)	12.4% (14)	20.3% (40)	12.1% (39)
Secondary/college	26.2% (166)	25.7% (29)	26.4% (52)	26.3% (85)
Graduate	43.4% (275)	45.1% (51)	37.6% (74)	46.4% (150)
Others	15.6% (99)	16.8% (19)	15.7% (31)	15.2% (49)

*Self-rated health*				
Poor	14.0% (89)	16.8% (19)	15.2% (30)	12.4% (40)
Good or better	86.0% (544)	83.2% (94)	84.8% (167)	87.6% (283)

*Social environment motivator*				
Not at all	22.9% (145)	50.4% (57)	20.3% (40)	14.9% (48)
Friends	29.8% (189)	23.0% (26)	33.5% (66)	30.0% (97)
Family	47.3% (299)	26.6% (30)	46.2% (91)	55.1% (178)

*Physical functioning limitations*				
No limitations	65.9% (417)	27.4% (31)	54.8% (108)	86.1% (278)
Some limitations	34.1% (216)	72.6%% (82)	45.2% (89)	13.1% (45)

*Note.* Statistics indicate the percentage (%) and number (*n*) of participants. Minimum PA = at least 75 minutes of physical activities per week. Moderate PA = 150 minutes of physical activities per week. Vigorous PA = 300 minutes of physical activities or more per week.

**Table 3:** Vigorous and moderate physical activity levels.

PA levels			Sports participants		Exercise participants		RCAs participants	
			*n* = 117	(%)	*n* = 206	(%)	*n* = 310	(%)
Vigorous PA	Days	1	10	8.54	15	7.28	22	7.10
		2	12	10.25	27	13.11	34	10.97
		3	33	28.21	58	28.16	77	24.84
		4	30	25.64	52	25.24	73	23.55
		5	17	14.53	24	11.65	60	19.35
		6	11	9.41	21	10.19	33	10.65
		7	4	3.42	9	4.37	11	3.54
	Minutes	0–30	20	18.80	39	18.93	26	8.39
		31–60	50	42.73	83	40.29	135	43.54
		61–90	25	21.37	62	30.10	85	27.43
		91–120	14	11.96	17	8.25	54	17.42
		121+	6	5.13	5	2.43	10	3.22

Moderate PA	Days	1	8	6.84	11	5.34	13	4.20
		2	12	10.26	41	19.90	48	15.48
		3	27	23.08	54	26.21	69	22.26
		4	30	25.64	47	22.42	71	22.90
		5	25	21.37	29	14.04	54	20.65
		6	10	8.55	14	6.80	28	9.03
		7	5	4.24	10	4.85	17	5.48
	Minutes	0–30	35	29.90	37	17.96	13	4.20
		31–60	46	39.32	81	39.32	94	30.32
		61–90	17	14.53	70	33.98	113	36.45
		91–120	13	11.11	12	5.82	63	20.32
		121+	6	5.13	6	2.92	27	8.71

**Table 4:** Mean, standard deviation, rank, *F*-statistic, and effect of exercise, sports, and RCAs participants.

	Exercise participants			Sports participants			RCAs participants			Statistical results	
	M	SD	Rank	M	SD	Rank	M	SD	Rank	*F* (df_1_ = 2; df_2_ = 630); *P*	Effect
Affiliation	3.85	0.37	3	3.96	0.37	5	3.96	0.29	3	*F* = 5.689; **p=0.004**	0.346
Appearance	3.66	0.36	14	3.81	0.41	10	3.86	0.38	10	*F* = 5.316; **p=0.005**	0.054
Challenge	3.69	0.42	13	3.75	0.37	12	3.88	0.33	8	*F* = 1.917; *p*=0.148	−0.030
Competition	3.77	0.40	8	3.84	0.32	9	3.90	0.38	6	*F* = 1.932; *p*=0.146	0.073
Enjoyment	3.82	0.47	5	3.98	0.40	3	4.05	0.35	2	*F* = 8.761; **p=0.000**	0.235
Health Pressures	3.80	0.37	6	3.88	0.32	6	3.85	0.36	11	*F* = 3.784; **p=0.031**	0.034
Ill-Health Avoidance	3.76	0.47	9	3.80	0.34	11	3.87	0.47	9	*F* = 6.564; **p=0.002**	0.200
Nimbleness	3.69	0.42	12	3.72	0.39	13	3.82	0.40	13	*F* = 1.123; *p*=0.326	0.045
Positive Health	3.95	0.27	1	3.99	0.29	2	3.95	0.37	4	*F* = 4.164; **p=0.016**	0.091
Revitalization	3.74	0.38	10	3.88	0.32	7	3.83	0.40	12	*F* = 1.740; *p*=0.178	0.025
Social Recognition	3.73	0.47	11	3.67	0.41	14	3.78	0.38	14	*F* = 1.743; *p*=0.176	−0.011
Strength and Endurance	3.78	0.39	7	4.10	0.39	1	3.92	0.37	5	*F* = 3.584; **p=0.038**	0.097
Stress Management	3.93	0.44	2	3.85	0.40	8	4.12	0.36	1	*F* = 3.964; **p=0.026**	0.143
Weight Management	3.84	0.30	4	3.97	0.32	4	3.89	0.48	7	*F* = 5.486; **p=0.004**	0.183

## Data Availability

The SPSS data used to support the findings of this study are available from the corresponding author upon request.
